# Case report: Re-emerging significance of surgical embolectomy in pulmonary embolism

**DOI:** 10.1016/j.amsu.2018.11.010

**Published:** 2018-12-03

**Authors:** Dana F. Kay, Jeko M. Madjarov, Bradley C. Tenny

**Affiliations:** Atrium Health-Carolinas Medical Center, North Carolina, USA

**Keywords:** Pulmonary embolus, All treatment strategies, Surgical embolectomy, Angiovac, (ECMO) Extracorporeal membrane oxygenation

## Abstract

**Introduction:**

Massive pulmonary embolus (PE) is associated with a high mortality if not treated aggressively. Treatment classically includes thrombolysis, catheter embolectomy, or open surgical embolectomy. This is the case report of a 38-year-old female presenting with massive PE three weeks status post gastric sleeve resection.

**Presentation of case:**

38-year-old female status post gastric sleeve resection presented to the emergency department with acute onset shortness of breath and dizziness. Computed Tomography (CT) chest angiography showed extensive PE with pulmonary artery saddle embolus, and an enlarged right ventricle suggesting strain. Her treatment consisted of anticoagulation, AngioVac suction embolectomy, EKOS catheter thrombolysis, fragmentation with catheter, extracorporeal membrane oxygenation (ECMO), and lastly surgical embolectomy due to refractory clinical course.

**Discussion:**

This case report details the natural history of a complex massive pulmonary embolism presentation requiring multiple catheter-based measures, ECMO initiation, and subsequent surgical embolectomy.

**Conclusion:**

This case report should serve as encouragement for early adoption of surgical therapy in pulmonary embolism cases where patients present with a complex presentation.

## Highlights

• Young female s/p abdominal surgery presented to the hospital for treatment of a massive pulmonary embolus.
• She underwent catheter-based thrombectomy and thrombolysis in the cardiac catheterization suite.
• Her condition worsened while attempting to come off bypass post thrombolysis warranting transition to ECMO.
• She was stabilized, transitioned to the ICU but continued to deteriorate necessitating surgical embolectomy.
• Surgical embolectomy resulted in removal of large pulmonary thrombus (see photos), and significant improvement in clinical condition.

## 1 Introduction

**Image 1:**
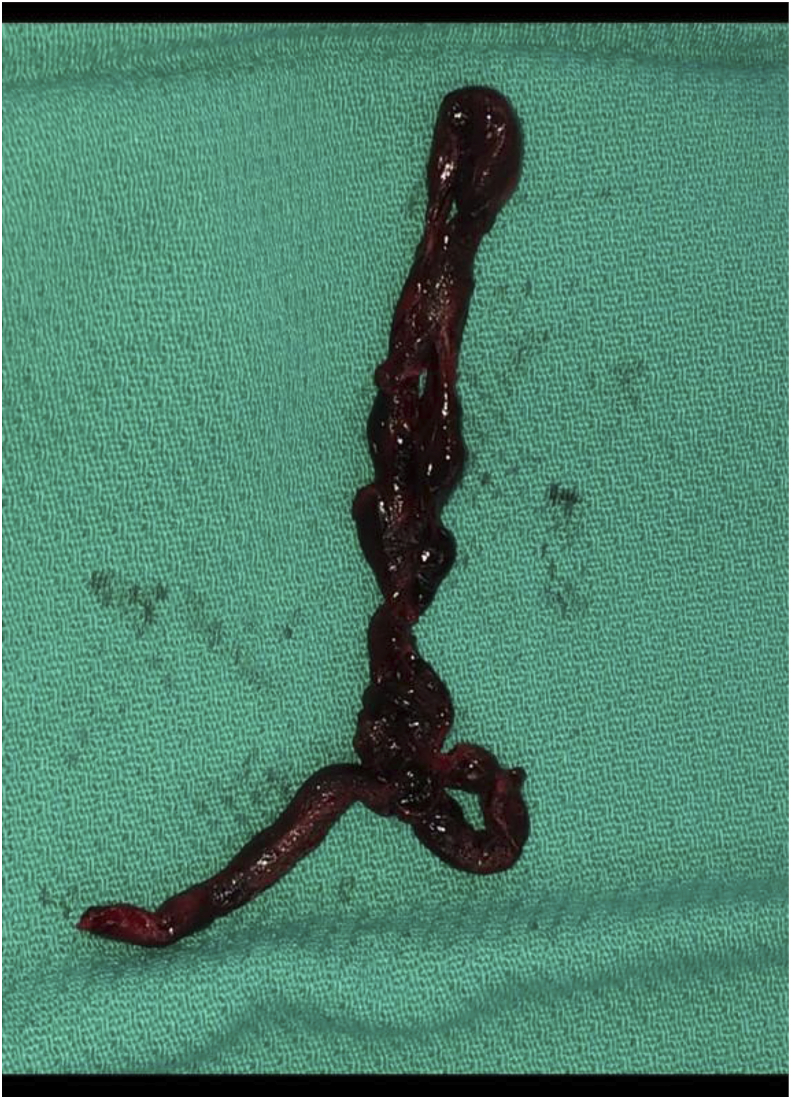
Thrombus that was originally extending from the RV into both the proximal pulmonary artery branches.

**Image 2:**
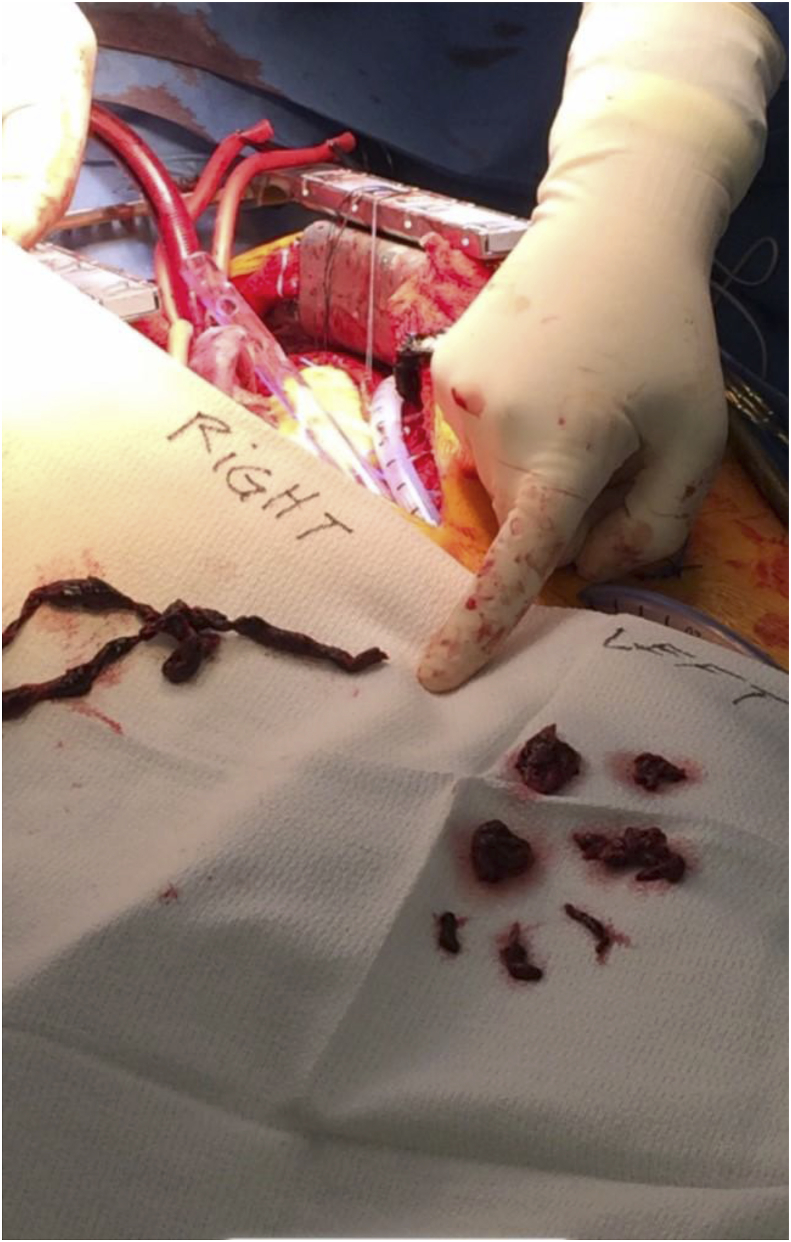
Thrombus retrieved from the right and left pulmonary arteries.

Massive pulmonary embolus is associated with a high mortality if not treated aggressively. The most effective treatment of pulmonary embolism is strongly debated. There are many guidelines, algorithms, opinions, but prospective studies are few**.** Treatment classically includes thrombolysis, catheter embolectomy, or open surgical embolectomy. This is the case report of a 38-year-old female presenting with massive PE three weeks status post gastric sleeve resection. The purpose of this case report is to illustrate the importance and effectiveness of surgical embolectomy by presenting a scenario where a massive pulmonary embolism was refractory to multiple catheter-based therapies. As new therapies are developed recognition and consideration for surgery should not be neglected. In our case report we present a patient who presented with a massive PE that underwent AngioVac suction embolectomy, EKOS catheter thrombolysis, fragmentation with catheter, ECMO, and lastly surgical embolectomy (see Image 1, Image 2).

## 2 Presentation of case

A 38-year-old female status post failed lap band surgery for morbid obesity that was now three weeks after gastric sleeve resection presented to the emergency department late in the evening with acute onset shortness of breath and dizziness. She had mild tachycardia and was normotensive at presentation. CT chest angiography showed extensive PE with pulmonary artery saddle embolus, and an enlarged right ventricle suggesting strain.

Initiation of anticoagulation was appropriately undertaken. Echocardiogram the following morning showed large mobile mass in the right atrium extending across the tricuspid valve into the right ventricle. Right ventricle pressure was noted at 35 mmHg. Furthermore, the right ventricle was dilated and hypokinetic. Systemic thrombolysis was felt to be unlikely successful given the large size of thrombus in transit of the pulmonary artery.

The decision was then made for catheter based thrombectomy with AngioVac; Angiovac is a catheter-based treatment option that uses a vacuum tipped catheter to suck out and remove the intraluminal clot. In the operating room prior to the AngioVac procedure a TEE was performed again to ascertain the characteristics of the problem, but the TEE now demonstrated that the RA thrombus had embolized with a resultant increase in the patient's HR and decrease in blood pressure. The patient was put on cardiopulmonary bypass and the AngioVac therapy was then quickly initiated. Of note, the patient was intubated and received general anesthesia for the procedure. There was notably only modest amounts of thrombus extracted from the main pulmonary artery. The interventional cardiologist then elected to break up the pulmonary artery clot by manual manipulation with a pigtail catheter. Next, an EKOS catheter, a catheter utilized for “localized” thrombolytic transfusion, was then placed to both the pulmonary arteries for tPA (alteplase) infusion. There was noted improvement of hemodynamics, and so the patient was separated from cardiopulmonary bypass, but quickly became hypotensive with electrocardiogram changes. The decision was then made to transition to ECMO, which was successfully undertaken.

Post procedure the patient was taken to the cardiothoracic intensive care unit for close monitoring with hopeful improvement with continued tPA infusion. Over the course of the next 8 h she had increasing pressor requirements and was ultimately taken for salvage pulmonary embolectomy via median sternotomy where the remaining large clot burden was evacuated from the bilateral pulmonary tree (see figures/pictures). ECMO decannulation was done at the time of sternotomy and the chest was left open with wound VAC placement. Wound Vac placement and being left open were to allow for the continued monitoring of acute surgical blood loss.

The following day she developed abdominal compartment syndrome, presumably due to bleeding into the peritoneal cavity, necessitating exploratory laparotomy and celiotomy. The abdominal wound was also left open, placed to VAC, and subsequently closed two days later. The day after abdominal closure the chest was closed with pectoral flaps.

The rest of her hospital stay was uneventful. She was noted to have remained in house 10 days since arrival to the hospital. The day prior to discharge to rehabilitation a repeat echocardiogram showed that the right ventricular pressure decreased to 29 mmHg with normal size and systolic function.

## 3 Discussion

This case report details the natural history of a complex massive pulmonary embolism presentation. To our knowledge it is the only present case report that details the use of catheter-based thrombolysis, catheter-based embolectomy, and surgical embolectomy in one whole article. In our literature search we found the most similar case report that details the joint use of catheter-based embolectomy and thrombolysis that was successful [2], however in our presented case the patient did not grossly benefit from the catheter-based therapies thus necessitating surgical embolectomy.

In this case the ineffectiveness of the catheter-based therapies was most likely multifactorial. The patient's clot burden was extensive with the clot extending from the pulmonary artery into the right atrium on initial echocardiogram. Additionally, catheter-based therapy has reported operator difficulty and can make for incomplete treatment or removal of clot [3].

In such, we advocate for earlier consideration for surgical embolectomy in the setting of massive pulmonary embolism. Awaiting alternative treatment success can greatly impact the success of subsequent surgery given the continued strain on the heart if said treatments are unsuccessful. In support of this view point there is readily available research detailing the greater efficacy, and lower complication rate in surgical embolectomy patients versus repeat thrombolysis. For example, the research article put forth by Azari and co. demonstrated that there was a statistically significant decrease in mortality in patient's who were assigned surgical embolectomy over thrombolytic therapy in treating acute massive pulmonary embolism. Furthermore, this comparative cohort study demonstrated statistically improved right sided heart pressures in the surgical embolectomy arm [4].

Putting this to practice would appear as consideration for emergent surgical embolectomy in the setting of a refractory condition to thrombolysis, or in the setting of significant clot burden and severe hemodynamic instability; both of which our patient suffered from. In our patient's case, her condition remained decompensated, and once weaned from the cardiopulmonary bypass, post catheter directed thrombolysis, she began to have further hemodynamic compromise necessitating ECMO. If consideration for surgical embolectomy was taken at this time of repeat decompensation her improvement may have very well been hastened.

## 4 Conclusion

Surgical embolectomy was a safe and effective intervention for our patient presenting with massive pulmonary embolus. In addition, it was the only therapeutic intervention that changed our patient's quickly worsening prognosis. Our patient received both catheter thrombolysis and catheter embolectomy, but inevitably required surgery due to a deteriorating condition. This case report serves as an excellent reminder of the utility of surgical embolectomy in massive pulmonary embolus, and we hope that it encourages the earlier adoption of surgical therapy in cases where patients present with a complex presentation with refractory presentation to initial thrombolysis.

## Ethical approval

Institutional review board approval obtained: IRB File # 03-18-09EX.

## Sources of funding

Self-funded.

## Author contribution

Dana Kay-Author.

Bradley Tenny- Co-author.

Dr. Jeko Madjarov- Co-author.

## Conflicts of interest

There were no conflicts of interesting in writing of this case report. Case report was author/institutionally (Sanger Heart & Vascular Institute) funded.

## Research registry number

None.

## Guarantor

Dr. Jeko Madjarov.

## Provenance and peer reviewed

Not commissioned, peer reviewed.

## Patient consent

Patient consent obtained.

## Appendix A Supplementary data

SCARE checklist-case report SCARE checklist-case report

## References

[2] Blair J. (2017). Transcatheter therapy for a large mobile right atrial thrombus and massive pulmonary embolism. J. Invasive Cardiol..

[3] Sobieszczyk P. (2012). Catheter-assisted pulmonary embolus. Circulation.

[4] Azari A. (2015). Surgical Embolectomy versus thrombolytic therapy in management of acute massiv e pulmonary embolism: short and long-term prognosis.

